# Measurement of Water Uptake and States in Nafion Membranes Using Humidity-Controlled Terahertz Time-Domain Spectroscopy

**DOI:** 10.1021/acssuschemeng.4c01820

**Published:** 2024-05-08

**Authors:** George A. H. Ludlam, Sam J. P. Gnaniah, Riccardo Degl’Innocenti, Gaurav Gupta, Andrew J. Wain, Hungyen Lin

**Affiliations:** † Department of Engineering, Lancaster University, Lancaster LA1 4YW, U.K.; ‡ National Physical Laboratory, Hampton Road, Teddington, Middlesex TW11 0LW, U.K.; □ School of Electronic Engineering and Computer Science, Queen Mary University of London, London, E1 4NS, U.K.

**Keywords:** proton-exchange membranes, membrane hydration, terahertz spectroscopy, water states

## Abstract

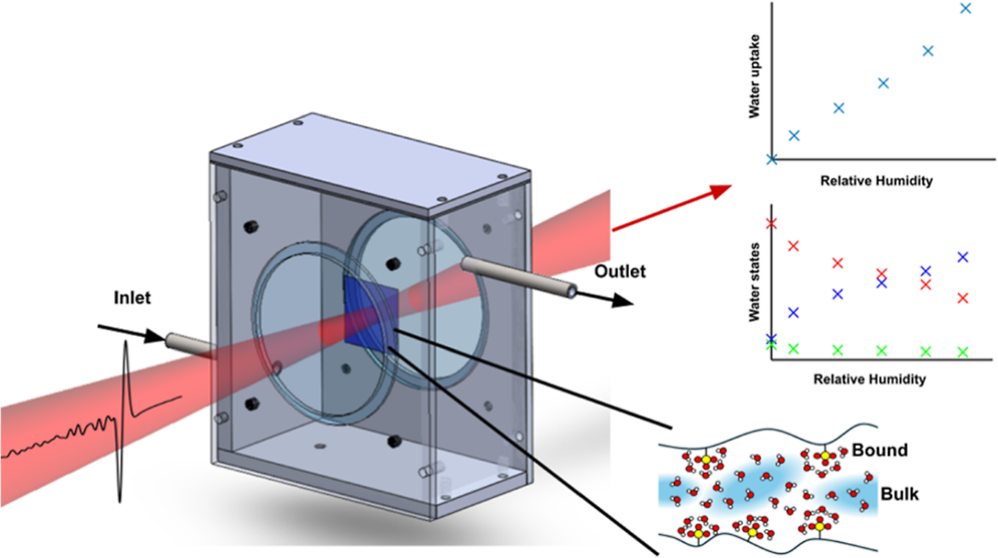

Perfluorinated sulfonic acid ionomers are well known for their unique water uptake properties and chemical/mechanical stability. Understanding their performance–stability trade-offs is key to realizing membranes with optimal properties. Terahertz time-domain spectroscopy has been demonstrated to resolve water states inside industrially relevant membranes, producing qualitatively agreeable results to conventional gravimetric analysis and prior demonstrations. Using the proposed humidity-controlled terahertz time-domain spectroscopy, here we quantify this detailed water information inside commercially available Nafion membranes at various humidities for direct comparison against literature values from dynamic vapor sorption, differential scanning calorimetry, and Fourier transform infrared spectroscopy on selected samples. Using this technique therefore opens up opportunities for rapid future parameter space investigation for membrane optimization.

## Abstract 2

Humidity-controlled terahertz time-domain spectroscopy to extract water uptake and states in membranes used in hydrogen fuel cells and electrolyzers to enable greater material understanding and optimization.

## 1 Introduction

**Figure 1:**
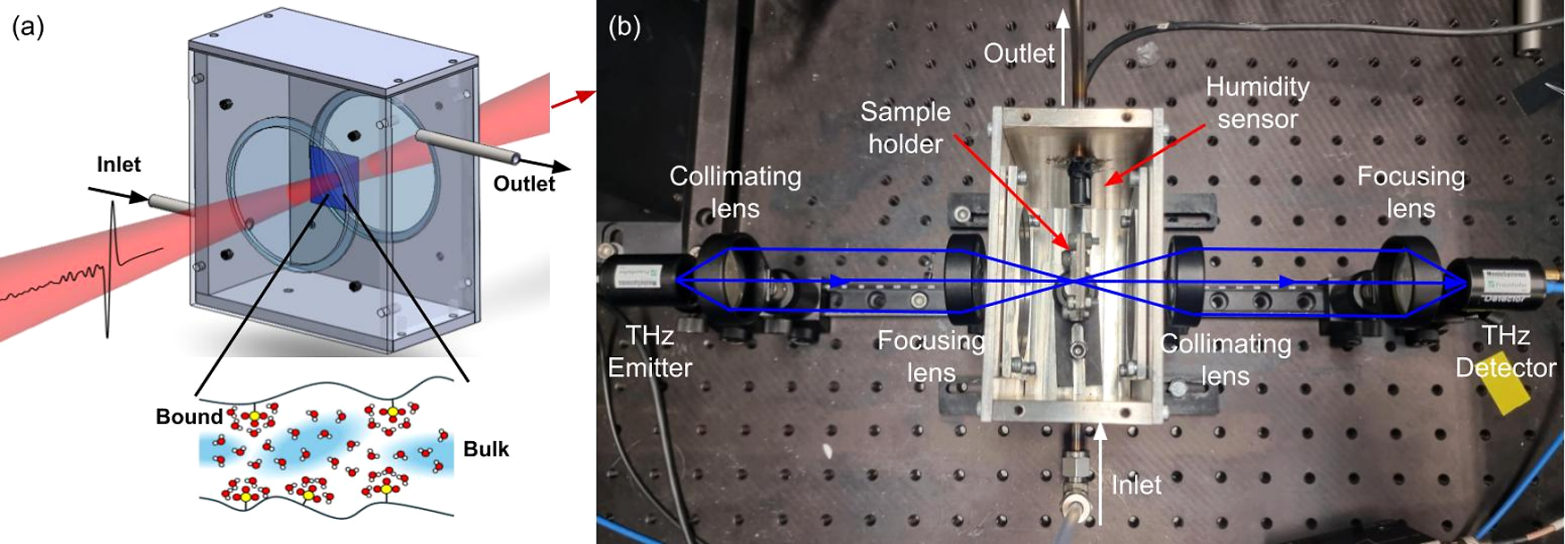
(a) Concept diagram of the proposed measurement to probe membrane water states. (b) Experimental setup using THz-TDS shown with the chamber lid removed.

Perfluorinated sulfonic acid (PFSA) ionomers are a common class of materials well known for their unique ionic conductivity and chemical–mechanical stability, thus widely used as membranes inside electrochemical devices^1−3^ or as sensors and actuators.^4^ These synthetic polymers consist of a semicrystalline, chemically inert, and hydrophobic polytetrafluoroethylene backbone with side groups terminated with hydrophilic sulfonate groups.^5^ The ionic conductivity of these membranes depends highly on the level of hydration since hydrophilic domains can combine to form interconnected ion conducting channels,^5−9^ underpinning transport mechanisms such as Grötthuss hopping, electro-osmosis, and back diffusion.^5,10−12^ The unique morphology of PFSA also gives rise to a variety of environments where water can exist, thus resulting in multiple different water states (Figure 1a) governed by a combination of geometric factors and intermolecular interactions. In particular, water within these hydrophilic domains can exist in 3 states: bound water (strongly hydrogen bonded and predominantly bound to the hydrophilic sulfonate groups),^10,13^ bulk water (weakly hydrogen bonded, exhibiting co-operative reorganization of hydrogen bonds),^13,14^ and free water (not hydrogen bonded). Due to the importance of hydration in terms of the performance of PFSAs, prevailing methods have been used for characterizing hydration such as small-angle X-ray scattering spectroscopy,^15−17^ neutron scattering and imaging,^18−22^ nuclear magnetic resonance,^23−26^ microwave dielectric relaxation spectroscopy,^10,27^ Fourier transform infrared (FTIR) spectroscopy,^28−32^ dynamic vapor sorption (DVS),^33,34^ differential scanning calorimetry (DSC),^35−42^ and Raman spectroscopy.^43−46^ These techniques have been used to study different aspects of membrane hydration such as structure,^15−17,22,30,47−50^ diffusion,^23,24,33,34,43,49,50^ and proton conduction.^15,25,27−29,36−38^ Terahertz time-domain spectroscopy (THz-TDS) is a rapid, noninvasive, and contactless technique, which has previously shown sensitivity to water^51−53^ and in particular water uptake (WU) and water states in PFSAs.^14,54^ The terahertz frequency region is of interest as it contains information on the reorientation dynamics of water, with contributions from bulk water relaxation at ∼18 GHz^10,13^ and free water relaxation.^13,14,54^ As terahertz radiation is highly sensitive to these relaxations, efforts have been made to quantify these water states in ambient environments.^14,54^ Given that these membranes will inevitably operate under varying humidities, in this work, we explore the possibility of extracting detailed information on WU and water states within commercially available Nafion membranes under controlled environments.

## 2 Materials and Methods

### 2.1 Samples

Membranes used include different grades of commercial Nafion (117, 211 and XL) (Fuel Cell Store, TX, USA) at nominal thicknesses of 183, 25, and 27 μm, respectively. Membranes were cut into 3 cm × 3 cm samples and pretreated by boiling in 3% H_2_O_2_, submersion in boiling deionized (DI) water, then boiling in 0.5 M H_2_SO_4_, and finally submersion in DI water at ambient conditions (1 h for each step). Three repeats for each type of membrane were prepared for each experiment.

Discrete levels of Nafion 117 WU were achieved by placing the pretreated membranes in sealed containers containing saturated salt solutions for 24 h to reach equilibrium. This is to control the relative humidities (RHs) of air surrounding the membrane for controlled hydration.^10^ The solutions used and RHs measured using a TP50 hygrometer (ThermoPro, USA) are listed in Table 1.

**Table 1:** Conditions for Membrane Hydration and the Expected WU

saturated salt solution	measured RH (%)	expected Nafion 117 WU (wt %)^55^
magnesium chloride	38–40	7.7
potassium carbonate	47	8.6
sodium chloride	74–78	13.1
DI water	100	26.0

### 2.2 Humidity Chamber

A bespoke humidity chamber was designed and realized to retrofit to the sample portion of the terahertz beam path in the THz-TDS setup. This step was necessary to minimize moisture exposure to the terahertz optics and devices to avoid material degradation.^52^ In particular, the humidity chamber was positioned within the focused region of the terahertz beam path as shown in Figure 1 and has welded pipes for inlet and outlet airflow, as well as two z-cut quartz windows for terahertz beam propagation.^56^ In principle, other highly terahertz transparent window materials could also be used such as high-density polyethylene and high-resistance silicon.^57^ The length of the chamber was designed with the windows being 28.5 mm from the sample to avoid etalon reflections within the 100 ps terahertz measurement window. This can be confirmed in Figure S1, which shows that the presence of the chamber has a negligible effect on the measurement. Humidified air was supplied to the chamber via the gas inlet and was prepared externally where dry compressed air was mixed with saturated air, which had been passed through a homemade bubble humidifier. The level of humidification was controlled by changing the flow rates of dry and hydrated air, which were controlled using mass flow controllers (Alicat, USA) connected to a PC via a breakout box for flow control networking. The total gas flow rate was set to 1 standard liter per minute, and the ratio of dry/wet gas was controlled using a PID controller within LabVIEW. Figure S2 shows the overall system setup. Humidity was measured inside the chamber as shown in Figure 1 using a T9602 polymer capacitance humidity sensor (Amphenol, USA) with a specified accuracy of ±2% at 20–80% RH and up to ±3.5% at 0–20% and 80–100% RH. As the chamber has a volume of 0.72 L, this results in a residence time of ∼43 s. Humidities were maintained to within ±0.1% of the measured RH, which took 10–20 min to reach steady state with the exception of 0% RH, which took up to 2 h. In general, based on the RH sensor readings, the chamber was able to operate between ∼0 and 85% RH consistently, above which, for example, at 90% RH set point, variations were observed as the temperature was not controlled. The lack of temperature control is a limitation of this system as it affects membrane WU^5,58^ and achievable RH. However, temperature was recorded for all experiments and varied between 20.7 and 25 °C.

### 2.3 Terahertz Time-Domain Spectroscopy

Transmission terahertz spectroscopy was performed using a commercial THz-TDS setup (TERA K15, Menlo Systems, Germany), as shown in Figure 1. Nafion 117 hydrated with saturated salt solutions was measured in free space without the humidity chamber present at ambient environmental conditions, and for each measurement, 20 averages were acquired. For chamber measurements, these were acquired at decreasing measured humidities of 90, 70, 50, 30, 10, and 0% RH at steady state for 2 h from a prehydrated state at 100% RH. To reduce the effect of laser jitter, a reference measurement was always acquired in the same environment as the sample but without the sample being present. Specifically for the chamber, the reference data was acquired at the same humidities as the sample to remove discrete water vapor absorption lines as shown in Figure S1, which would otherwise interfere with subsequent data analysis. Figure S2 further shows the increasing strength of these absorption lines with increasing humidity.^59^

### 2.4 Data Analysis

Prior studies have shown how macroscopic WU can be determined in hydrated membranes where an equivalent model of hydrated membranes is arranged as a dry membrane and a uniform layer of water thickness.^14^ As the dielectric properties of membranes at different WUs can be described using effective medium theory, here we assume a simple, linear mixing model relating the effective frequency-dependent absorption and the volume fraction of water in the system^60^ as shown in eq 1
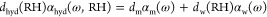
1where α is the absorption coefficient, ω is the angular frequency, and *d* is the thickness, subscripts hyd and m refer to hydrated and dry membrane, respectively, while w is the water contribution. By rearranging eq 1, the effective water thickness can be determined using eq 2
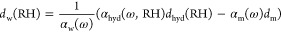
2

Absorption coefficients and thicknesses of dry and hydrated membranes are calculated from analyzing acquired waveforms using the previously developed parameter-based algorithm.^54^ In general, this algorithm models the transmitted electromagnetic wave through a dielectric slab with a complex refractive index *n̂*_s_ = *n*_s_(ω) – *ik*_s_(ω) at a normal angle of incidence in free space using plane-wave approximation shown in eq 3(54)
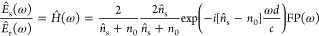
3where *Ê*_s_(ω) and *Ê*_r_(ω) are the Fourier transform of the sample and reference waveforms, respectively, *Ĥ*(ω) is the transfer function, *n*_0_ is the refractive index of air, *c* is the speed of light under vacuum, and *d* is the sample thickness. FP(ω) is the Fabry–Perot from multiple internal reflections given by eq 4
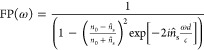
4

Iterative methods are then used to extract the optical parameters by minimizing the error between the modeled transfer function and the measured transfer function,^61−67^ commonly known as the objective function in optimization and expressed as
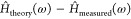
5

To ensure that the solver can arrive at physical solutions, a priori information on the dielectric response of the materials is included, which is valid for hydrated membranes as they are known to follow a double Debye response^10,14,54,68^ expressed as
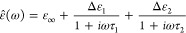
6where ε_∞_ is the infinite dielectric constant, Δε_1_ and Δε_2_ are the dielectric strengths of the bulk and free Debye relaxations, respectively, τ_1_ is the bulk relaxation time fixed at ∼8 ps,^10,14^ and τ_2_ is the free relaxation time. Bounds of the fitting parameters are shown in Table S1. The complex permittivity and absorption coefficient in turn are related to the complex refractive index using the following
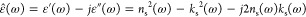
7
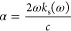
8

Using the absorption coefficient of liquid water^69^ together with the optical parameters and thicknesses from the solver, humidity-dependent effective water thicknesses and hence WU can be determined using eqs 2 and 9
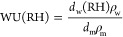
9where ρ_w_ and ρ_m_ are the densities of water (1 g/cm^3^) and Nafion (1.94 g/cm^3^^14^), respectively. Using the extracted dielectric strengths and WU, the proportions of bulk, bound, and free water states are then determined using eqs 10–12^13,14,54^
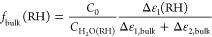
10
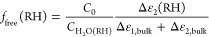
11
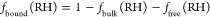
12where Δε_1,bulk_ and Δε_2,bulk_ are the dielectric strengths of bulk and free water relaxations for pure water, respectively, and *C*_0_ is the concentration of pure water (55.5 mol/L). The density of the hydrated membrane and the concentration of water within the membrane can be determined using eqs 13 and 14(14,54) respectively, where *M*_w_ is the molecular weight of water (18 g/mol)
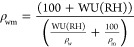
13
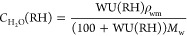
14

It should be noted that due to a relatively long measurement delay between sample and reference measurement to reach steady-state humidity, terahertz pulse drift is likely, and hence, the resultant phase is corrected by multiplying the transfer function by a phase shift term exp(-*i*Δ*t*ω),^70^ where Δ*t* corresponds to a small timing change (<15 fs) for selected measurements. As thin membranes are particularly susceptible to pulse shifts than their thicker counterparts, phase correction is applied to outlier phases which due to laser jittering deviate from an observed trend of approximately equal phase spacing between RHs. Given the amount of correction applied can affect the extracted membrane thicknesses, this amount is validated by comparing the resultant thicknesses against actual thickness using a micrometer taken immediately after each terahertz measurement. In particular, the thickness difference between the two modalities was generally less than 5% for thin membranes. To ensure high-quality fitting to the measurement for the robust extraction of the parameters, the fitting spectral range is taken up to 1 THz, above which water vapor absorption becomes increasingly dominant (see Figure S3). The choice over this spectral range also coincides with the rotational relaxation of water.^13,71,72^ All of the acquired terahertz measurements were processed using codes developed in Matlab (Mathworks, Inc., MA, USA).

For comparison against DSC data, conversion to respective water contents [H_2_O/SO_3_] was performed using the following
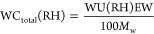
15
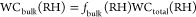
16
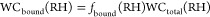
17where WC is the water content [H_2_O/SO_3_] and EW is the equivalent weight of Nafion (1100 g/mol).

### 2.5 Differential Scanning Calorimetry and Thermogravimetric Analysis

DSC was performed using a DSC Q2000 instrument (TA Instruments) using a temperature modulation mode. The same Nafion 117 samples were cut into small (2–5 mg) pieces and equilibrated at room temperature for at least 2 h prior to measurement either in DI water or at 85% RH in a humidity chamber. Samples were transferred rapidly into aluminum DSC crucibles with minimal (<1 min) exposure to the ambient atmosphere before sealing. The DSC cycling program comprised a negative temperature ramp from 20 to −90 °C at 2 °C/min cooling rate, followed by a positive temperature ramp up to 160 °C at 2 °C/min. A temperature modulation amplitude of ±1 °C every 60 s was used to reduce the problem of looping artifacts caused by supercooling effects. The instrument software Universal Analysis (UA) was used for the peak integration analysis of the thermogram, shown in Figure S4. The mass of freezable water was calculated by integrating the endothermic peak on the heating thermogram associated with ice melting (between −20 and 0 °C) and assuming that the enthalpy of freezing of water is 314 J/g.^37^ The total mass of water was calculated by integrating the broad endothermic peak associated with water evaporation (between 20 and 120 °C) and taking the enthalpy of vaporization of water to be 2258 J/g.^73^ For each condition, repeated measurements were performed on at least two samples.

Thermogravimetric analysis (TGA) was performed using a Q5000 IR instrument (TA Instruments). The Nafion 117 sample was cut into 5 mg pieces and equilibrated at room temperature in DI water for at least 2 h prior to analysis. Samples were dabbed with tissue paper to remove any excess water and transferred rapidly (<30 s transfer time) into aluminum TGA crucibles before sealing. A temperature ramp of 5 °C/min was employed from room temperature up to 200 °C, and the mass change at 150 °C was used to calculate the total water content. Repeat measurements were performed on at least two samples.

## 3 Results

### 3.1 Water Uptake

**Figure 2:**
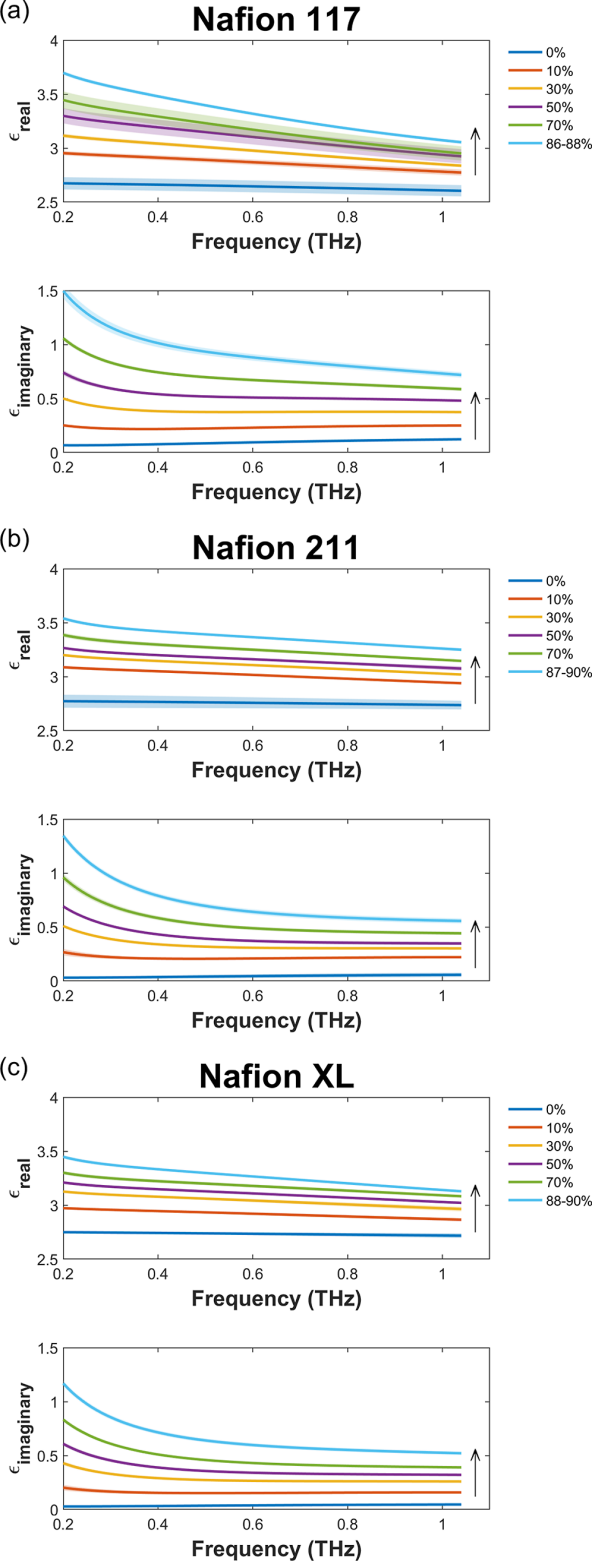
Real and imaginary parts of the complex dielectric permittivity of (a) Nafion 117, (b) Nafion 211, and (c) Nafion XL at different RHs.

**Figure 3:**
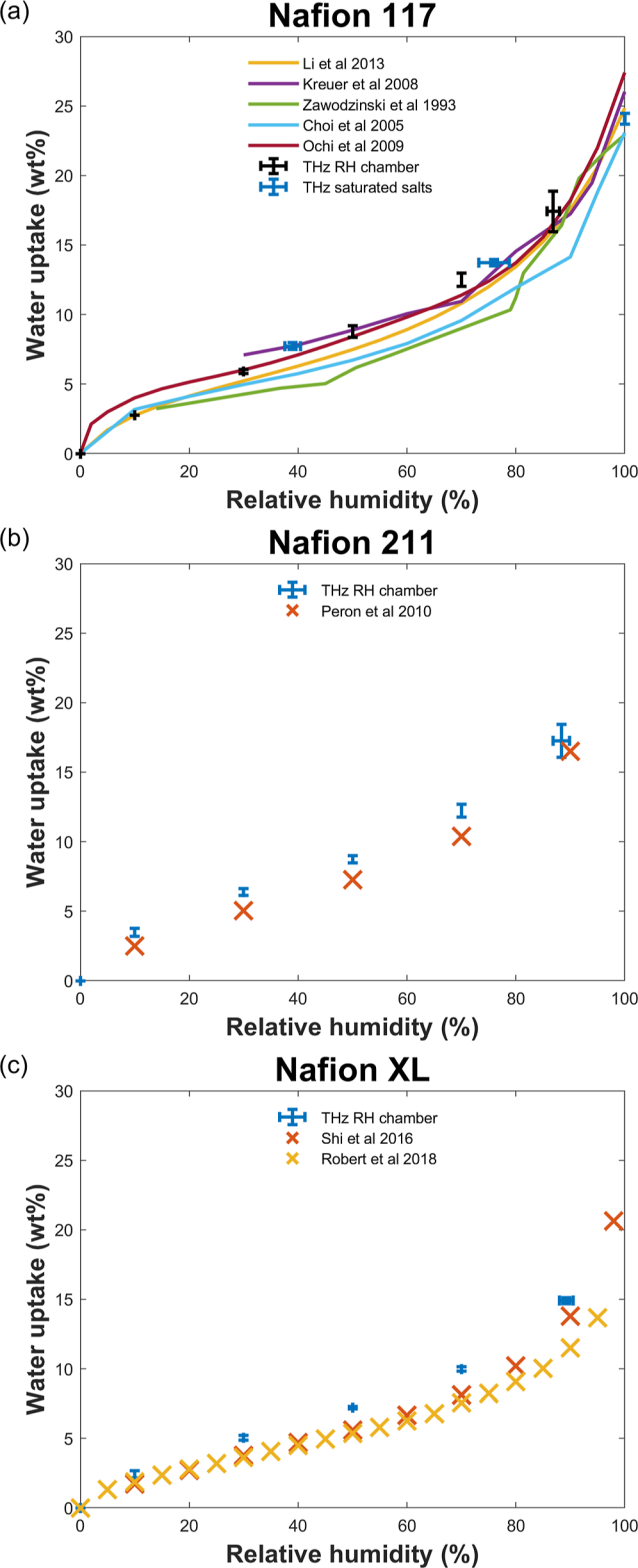
Isotherms of (a) Nafion 117, (b) Nafion 211, and (c) Nafion XL from THz-TDS against literature DVS values.

Figure 2 shows the extracted dielectric response for the three different Nafion membranes at measured RHs of 0, 10, 30, 50, 70, and 86–90%, and as expected, both the real and imaginary components of the complex dielectric permittivity increase with humidity.^10^ Fitting results are shown in Figure S5. Given the quality of the fits and a general agreement with the micrometer-measured thicknesses, the response therefore confirms the broad applicability of our data analysis algorithm.^54^ It also highlights that these data can be well described by using the double Debye response (eq 6) with τ_1_ fixed to the bulk water time constant at ∼8 ps. Such a value is also observed in pure water in the terahertz region^69,72,74,75^ as well as hydrated Nafion 117 using dielectric spectroscopy.^10^ These results therefore confirm that these hydrated membranes contain water molecules with reorientation dynamics similar to bulk water molecules.^10,14,54^ Using the extracted thicknesses and optical parameters of the hydrated membranes, effective water thicknesses were determined using eqs 2 and 9 to produce humidity-dependent WU as shown in Figure 3. These results are compared against literature values acquired using gravimetric-based DVS,^55,76−82^ where a general agreement between the nonlinear uptake profile is observed for the different Nafion membranes with small differences between the absolute WU values. The data obtained in the current study for Nafion 117 is additionally consistent with data at RHs controlled using salt solutions where our measurements are also in agreement with prior work that used cuvettes shown in Figure S6,^83,84^ which extends down to sub-GHz frequencies. The differences observed could possibly be due to variations in how the membranes have been pretreated and their thermal history. Furthermore, due to a lack of temperature control in the realized chamber, variations are also be expected. In the case of Nafion XL, these differences are additionally convoluted by the hysteresis^80^ where the only accessible literature data is related to sorption instead of desorption.^81,82^ These results therefore suggest that effective medium theory can be used to estimate the effective water thickness, which reduces to zero at 0% RH resulting in a zero WU in line with DVS where residual water is generally ignored.^5,82,85−87^ Here, we estimate the residual water that requires an elevated temperature for removal^86^ as the measured value at 0% RH, and while this is generally in agreement with literature values, our values are slightly lower.^5,88,89^

### 3.2 Water States

**Figure 4:**
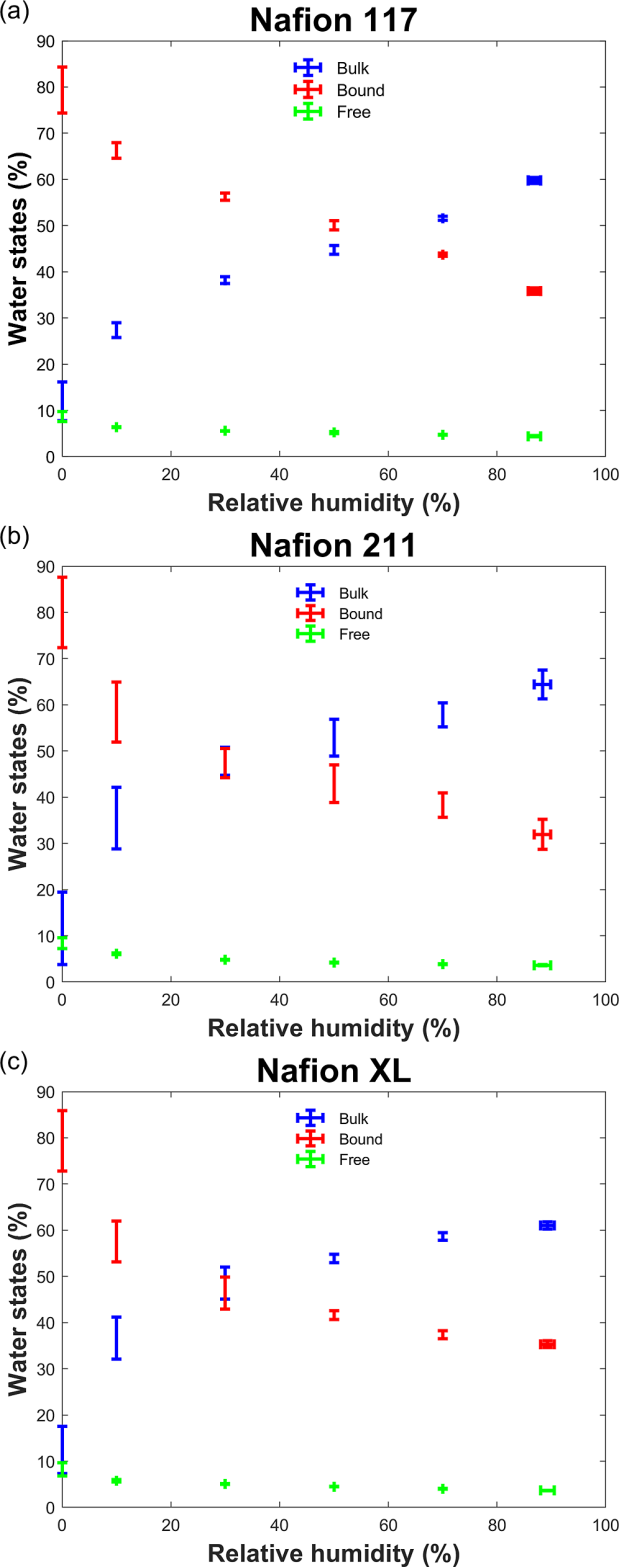
Humidity-dependent water states of (a) Nafion 117, (b) Nafion 211, and (c) Nafion XL.

Using the extracted model parameters from eqs 10 to 12 in a manner similar to previously,^13,14,54^Figure 4 shows the proportion of RH-dependent water states in Nafion membranes where error bars for thin Nafion 211 and XL are greater than for thicker Nafion 117 possibly due to laser jittering, material handling, as well as uncertainties associated with membrane residual water. As expected however, the relative proportion of bulk water does increase with increasing humidification, while a concomitant decrease in bound water is observed. This behavior is generally in qualitative agreement with understanding^5^ and observations made using other characterization methods (e.g., DSC^37^ and dielectric spectroscopy^10^), membrane systems,^13^ and prior work.^54^ In particular, bound water dominates at low RHs, and as the water activity in the membrane increases through humidification, the proportion of bound water contribution decreases in exchange for an increase in bulk water. The value of RH at which there is a crossover between bulk and bound water is different for membrane types and thicknesses. Specifically, the crossover point for Nafion 211 and XL occurs at ∼30% RH, lower than the ∼60% RH observed for Nafion 117. For Nafion 211, this may be associated with the higher WU^5,90^ resulting in a greater proportion of bulk water due to the membranes being dispersion casted as opposed to extruded.^91,92^Figures S7 and S8 further compares the water properties of membranes used in this work. In particular, WU did not show significant difference for varying membrane thicknesses, but bulk water proportions are reduced with thicker Nafion 117 compared to thinner Nafion 211, resulting in slightly lower proton conductivity at low RHs.^80,93^ At high RHs, however, bulk water proportions for Nafion XL and 117 are similar, resulting in similar proton conductivities^82,94^ but are ∼6% lower than Nafion 211. The reduction in bulk water within Nafion XL compared to Nafion 211 suggests that water domains have been disrupted under hydrophobic PTFE reinforcements, thus decreasing the ability of the membranes to accommodate water, consistent with a reduced WU seen in Figure 3 and consequently a reduced proton conductivity.^82,95^ This highlights that even though membrane thickness may play a role in the water properties, other factors such as heat treatment, reinforcements, and manufacturing conditions^5,54,93^ will inevitably need to be considered.

**Figure 5:**
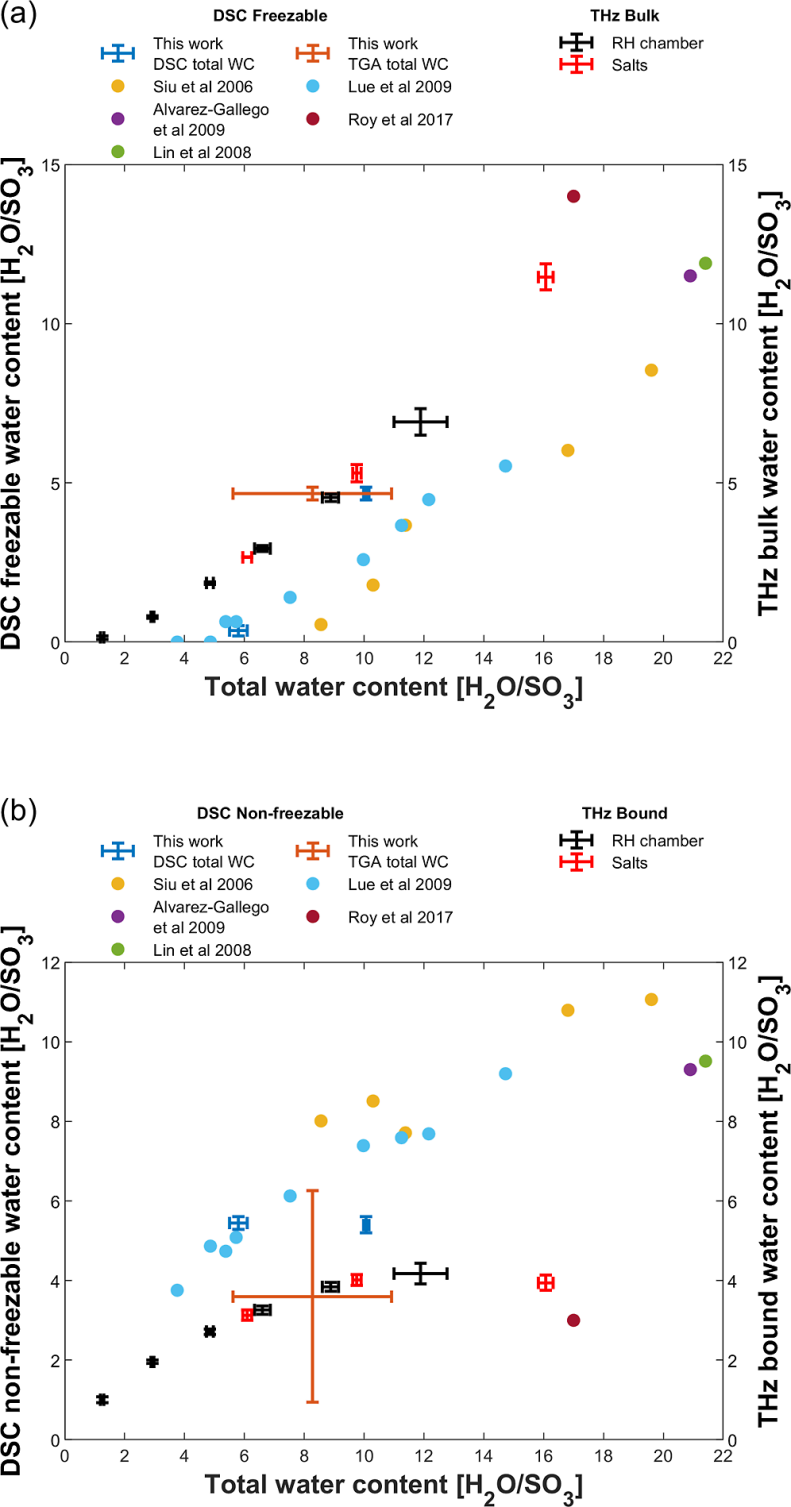
Nafion 117 water states comparison of (a) bulk and (b) bound states against DSC water states.^35,37−40^

DSC is routinely used for discriminating and quantifying freezable and nonfreezable water content in membrane systems,^35,37−42^ which can provide an indication of the water states. We therefore performed DSC measurements on the same Nafion 117 membrane used in terahertz measurements to compare the distributions of water states derived from these two techniques. Figure 5 shows the freezable/nonfreezable water content estimated from our DSC measurements against DSC literature values.^35,37−40^ Our DSC results are broadly consistent with the trend observed in the literature values, although we note that some differences are observed for the fully humidified sample, which exhibited a slightly higher freezable (and lower nonfreezable) water content compared to the literature. One possible reason for these differences is that we used the water evaporation peak in the DSC thermogram to estimate the total water content, while it is more common in the literature to calculate this by TGA. A difference may arise because the DSC calculation assumes the enthalpy of water vaporization to be the same in the membrane as it is for liquid water, which may contribute a significant source of error. To investigate this, we performed TGA on the fully humidified Nafion 117 to estimate the total water content and the associated data points in Figure 5. Importantly, the total water estimated by TGA was found to be slightly *lower* than that estimated by DSC, so this cannot account for the deviation between our nonfreezable water calculation and that reported in the literature. Hence, there must be additional experimental factors that are responsible for this discrepancy, and these are discussed in Section 3.3. Importantly, we note that the use of TGA to estimate the total water introduces considerably more error to the water state information compared to the use of DSC for this purpose, which highlights a potential weakness in using this approach for quantitative analysis. The reasons for the large error in the TGA-derived total water content is unclear, although this may relate to rapid equilibration of the fully hydrated Nafion samples with the ambient lab environment immediately prior to measurement, leading to different degrees of dehydration.

In order to compare terahertz bulk/bound against DSC freezable/nonfreezable water content, we converted terahertz water fractions from Figure 4 into their respective water content as described above, where a similarity between the respective trends can be observed consistent with other hydrophilic polymers.^96^ Despite this similarity, terahertz data generally reports a higher bulk water content than DSC freezable water for a given total water content (Figure 5a) possibly due to (1) bulk water fusion enthalpy being used to estimate freezable water content from DSC as opposed to a lower membrane-dependent value,^36^ which would increase the freezable water content; (2) total water content used to calculate water states from DSC is often independently measured gravimetrically,^35,38,39,41^ which can introduce considerable uncertainty; and (3) differences in the boundary between the water states being probed^96^ resulting in some of the nonfreezable water being incorrectly categorized as bulk water by the terahertz measurement. The latter difference arises from different physical parameters being measured, e.g., water fusion enthalpy by DSC as opposed to bulk water dielectric strengths by terahertz. While there is also some similarity between the trends between nonfreezable and bound water (Figure 5b), this similarity is less compared to the freezable/bulk water case. DSC is further prone to uncertainties from the independently measured water content convoluted by aforementioned factors that propagate into the respective water content calculation.

**Figure 6:**
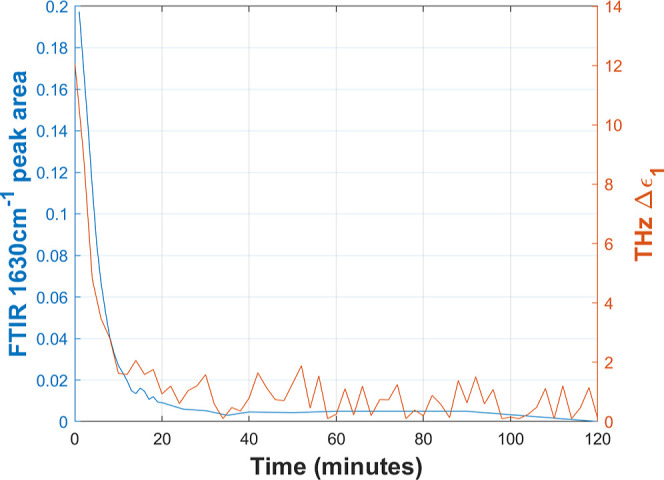
Terahertz bulk dielectric strength for comparison with literature FTIR peak area.^28^

FTIR spectroscopy can provide an alternative means to characterize water in PFSA membranes. In particular, Kunimatsu et al.^28^ demonstrated that there is a linear correlation between the band area of the peak at 1630 cm^–1^ (assigned to the HOH bending vibration of water molecules associated with SO_3_^–^ groups) and the membrane proton conductivity. To compare our terahertz measurement against the FTIR data reported by Kunimatsu et al.,^28^ we performed an equivalent experiment in which a 90% RH hydrated Nafion 211 membrane was dehydrated by continuously purging the chamber with dry air while acquiring terahertz response as a function of drying time. Figure 6 compares the rapid decay of the reported area under the 1630 cm^–1^ peak^28^ against the dielectric strength for bulk relaxation, which is associated with bulk water (see eq 6).^28^ These results show that during dehydration, water rapidly desorbs from the hydrated membrane under the driving force of osmosis, resulting in a rapid bulk water decay accompanied by an increase in bound water, while free water remains constant. Such an observation is consistent with prior studies of Nafion dehydration under ambient conditions.^14,54^ Comparing terahertz data against FTIR, a correlation can be observed, suggesting that the extracted terahertz bulk relaxation data may serve as a proxy for proton conductivity.

The distinction between bulk, bound, and free water in the terahertz regime ultimately stems from fundamental temperature-dependent water studies.^69,72,74,75^ There is a general understanding that bulk water corresponds to collective reorientational dynamics of the dipole moment with a resonance at ∼18 GHz or a relaxation time of ∼8 ps at room temperature resolvable at microwave^10,97^ and terahertz frequencies^69,72,74,75^ and confirmed by molecular dynamics simulations.^69^ This collective relaxation, however, is not Raman active as opposed to free water, which could be,^72^ with origins from the collisional relaxation inside the nonhydrogen bonding structure.^75^ The remaining water population according to eq 12 is assigned as bound water, which has been shown to contain both strongly and loosely bound water in solutions.^98^ DSC nonfreezable water is generally associated with strongly bound water,^10,98^ but this was shown to be higher than the total bound water determined from our terahertz data, indicating inconsistency between these measurements. This could be possibly due to fundamental differences in the measurement techniques resulting in differences in water states being probed in addition to the significant variations in DSC data, as discussed earlier. Dielectric spectroscopy could be an alternative to probe strongly bound water with microsecond relaxation time at ambient temperatures.^10^ Elucidating the different water states across different techniques is therefore subject to future investigation including the recently proposed environmentally controlled Raman spectroscopy.^99,100^

### 3.3 Practical Limitations

In spite of the challenges associated with comparing techniques that probe different parameters, we have observed a general agreement between the presented terahertz results against literature DVS, DSC, and FTIR data. However, it is also important to highlight some associated practical challenges. Firstly for the terahertz measurements presented here, a number of limitations should be considered in addition to those pointed out previously:^54^ (i) the terahertz measurements performed using the controlled humidity chamber results are desorption data only, thus will differ from sorption due to membrane hysteresis; (ii) no temperature control was implemented; thus, variations will exist in the quantified water states; (iii) there are practical challenges in maintaining high humidities (i.e., ≥90%) inside the humidity chamber, and this is the range particularly relevant to observe the steep WU increase; and (iv) increased uncertainties for thinner membranes (even though THz-TDS has sensitivity to dry polymeric films down to micron scales, thicknesses at least an order of magnitude greater than this are required for reliable characterization^57^). These factors are inevitably affected by sensor uncertainties, as some deviations from actual humidities can be expected. This is especially the case at 0% RH where some moisture is expected to remain.

The use of DSC to quantify the freezable water content can also be challenging, particularly when attempting this at different, controlled total water contents. For measurements using fully humidified membranes, the water freezing/melting events are easily detectable in the thermograms, but, as noted in Section 2.5, supercooling effects can lead to crystallization loops^101^ which may compromise the quantification. Such effects can be minimized by employing a slow temperature ramp and/or employing temperature-modulated DSC, in which a sinusoidal perturbation is superimposed on the linear temperature ramp. Humidity control is not typically possible using DSC instrumentation; therefore, performing DSC measurements on membranes at known levels of humidity below saturation is difficult to achieve accurately. In this work, the membranes were equilibrated at 85% RH in an environmental chamber, but as soon as the sample is removed from the controlled environment, re-equilibration with the ambient lab conditions will begin instantly. While steps were taken to minimize the ambient exposure time, it is very likely that the actual water content at the point of measurement will have decreased by an unknown amount. The impact of this will depend not only on the thickness of the membrane (thicker membranes are expected to re-equilibrate more slowly than thinner ones) but also on the type of crucible used in the DSC measurement (i.e., whether or not it creates an airtight seal). Furthermore, we noticed that the water freezing events in the DSC thermograms for the 85% RH samples were very weak, so measurements at lower total water content would be expected to be below the limit of detection (this was confirmed by the absence of any detectable freezing events in membranes equilibrated at ∼50% RH). Hence, DSC is limited to a relatively narrow range of total water contents, and even for the samples equilibrated at 85% RH, we expect considerable uncertainty (of the order of 20%) associated with the low signal-to-noise ratio and difficulties in selecting appropriate baselines for the peak integration.

In contrast to DSC, humidity control with FTIR during measurement is more straightforward,^28^ and the measurement is sensitive enough to detect very low amounts of water. The most significant challenge with FTIR, however, lies in the analysis and spectra interpretation, as the OH^–^ stretching and HOH bending IR modes are typically very broad and comprise multiple peaks. Deconvoluting these multicomponent bands by peak fitting is therefore challenging, and uncertainties arise as to the individual component band assignment. Moreover, quantitative FTIR spectroscopy is not recommended without appropriate calibration data, as absorbance does not necessarily scale linearly with analyte concentration, particularly in highly concentrated, strongly absorbing media like water.^102^

## 4 Conclusions

In this work, we have demonstrated the possibility of quantifying WU and states inside Nafion 117, Nafion 211, and Nafion XL membranes using the proposed humidity-controlled THz-TDS. This has produced WU data consistent with literature DVS values and water state trends in agreement with literature DSC and FTIR data. Even though we have probed PFSAs, without a loss of generality, the proposed technique is also applicable to anion-exchange membranes, where THz-TDS has also demonstrated sensitivity with results consistent with complementary small-angle X-ray and neutron scattering measurements.^103^ As an emerging technique, table-top based humidity-controlled THz-TDS can probe samples rapidly and nondestructively under controlled environments, thus opening up opportunities for future membrane testing. This will complement existing techniques to enable a greater material understanding and optimization of performance–stability trade-offs for a range of green technologies such as hydrogen fuel cells and electrolyzers underpinning a sustainable, green economy.

## Data Availability

Additional data sets related to this publication are available from the Lancaster University data repository https://doi.org/10.17635/lancaster/researchdata/665.

## Supporting Information Available

Chamber and free-space comparison, system photo, water vapor absorptions, DSC thermogram, fitting details, permittivity comparisons, and WU and WS comparisons (PDF)
