# Supplementary material for: Measurement of Water Uptake and States in Nafion Membranes Using Humidity-Controlled Terahertz Time-Domain Spectroscopy
Source: ACS Sustain Chem Eng. 2024 May 8;12(20):7924–34. doi: 10.1021/acssuschemeng.4c01820 (PMC11110106; doi:10.1021/acssuschemeng.4c01820)
Supplement: Supplementary file 1 — sc4c01820_si_001.pdf [file sc4c01820_si_001.pdf]

Supplementary information for

Measurement of water uptake and states in Nafion membranes using humidity-controlled terahertz time-domain spectroscopy

George A. H. Ludlam[1], Sam J. P. Gnaniah[2], Riccardo Degl'Innocenti[1,3], Gaurav Gupta[1], Andrew J. Wain[2], Hungyen Lin[1*]

[1]Department of Engineering, Lancaster University, Lancaster LA1 4YW, United Kingdom
[2]National Physical Laboratory, Hampton Road, Teddington, Middlesex, TW11 0LW, , United Kingdom
[3]School of Electronic Engineering and Computer Science, Queen Mary University of London, London, E1 4NS, United Kingdom

*h.lin2@lancaster.ac.uk Tel: +44(0)1524 593013

7 pages, 8 figures, 4 tables

THz-TDS with chamber

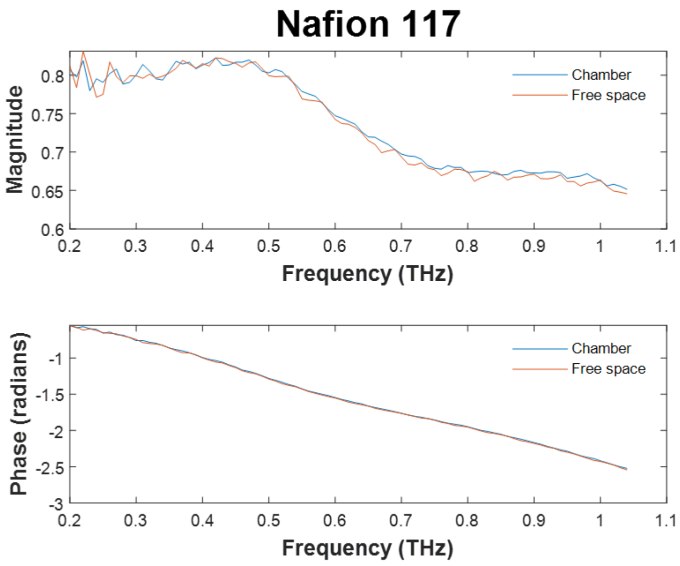

Figure S1 - Comparison of chamber and free space membrane magnitude and phase response under ambient conditions.

Humidistat setup

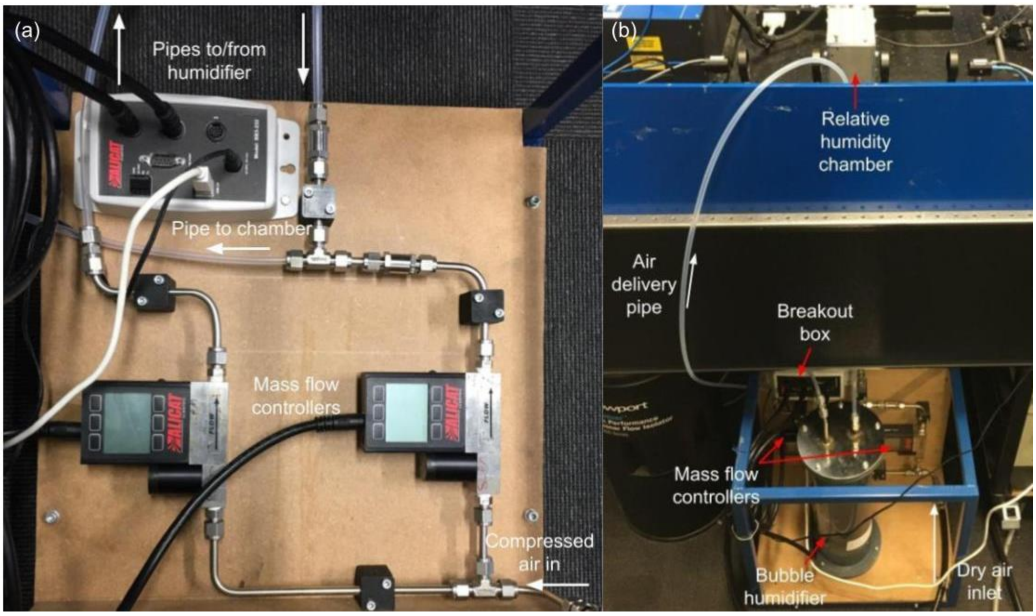

Figure S2 – (a) Pneumatic connections and (b) an overview of the system setup.

Water vapor absorption lines

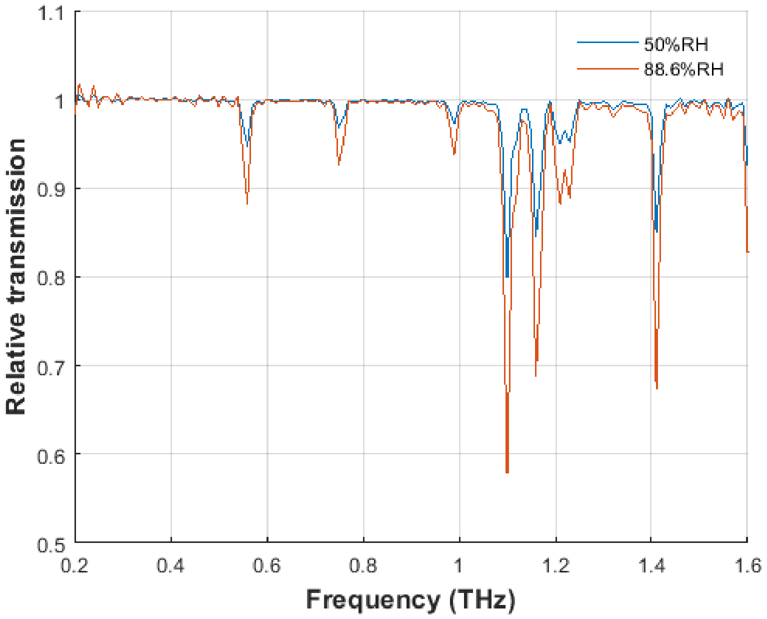

Figure S3 – Transmission showing water vapor absorption lines of chamber equilibrated at 50% RH and 88.6% RH, reference used was 0% RH.

Differential scanning calorimetry

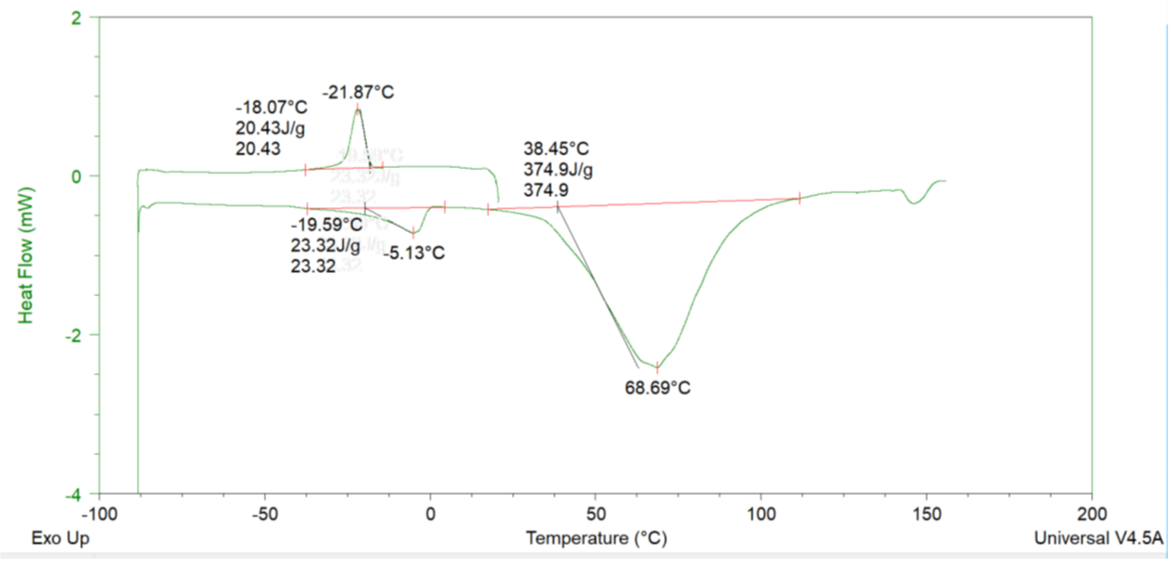

Figure S4 - DSC thermogram for a fully hydrated Nafion 117, exhibiting water freezing/melting events (peaking at -22 °C/-5 °C, respectively), and water vaporisation (peaking at 69 °C). The initial spike at the start of the test is an instrument startup artefact that is generally discarded.

Fitting range

Variable	Lower bound	Upper bound
ε_∞	2.2	3
$\Delta\varepsilon_1$	0.01	30
$\Delta\varepsilon_2$	0.01	2
τ_2 (ps)	0.08	0.4

Table S1 - Search range of selected fitted variables.

Fitting complex transfer function

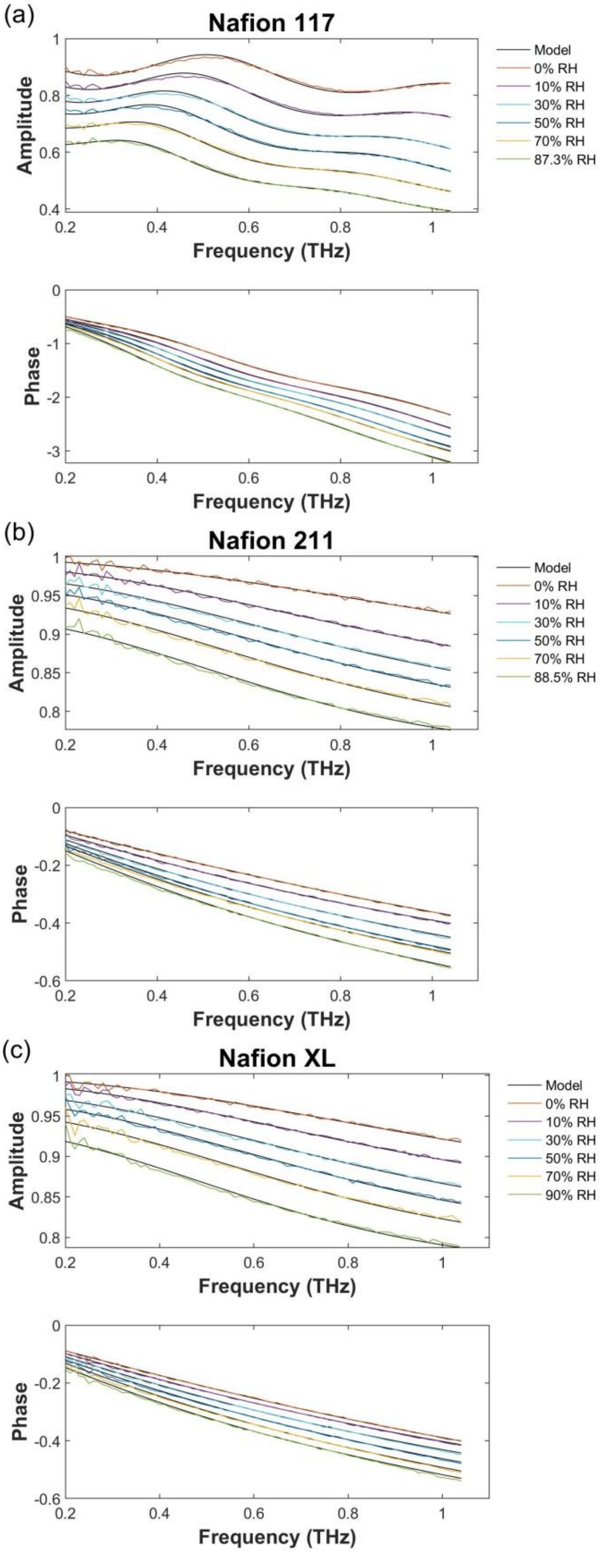

Figure S5 – Measurement and fittings of the complex transfer function for Nafion 117 (a), Nafion 211 (b) and Nafion XL (c) as function of RH

RH (%)	ε_∞	$\Delta\varepsilon_1$	$\Delta\varepsilon_2$	$\tau_2(ps)$
0	2.41	0.38	0.31	0.078
10	2.51	2.03	0.45	0.11
30	2.51	4.02	0.60	0.14
50	2.58	5.96	0.72	0.15
70	2.59	8.81	0.81	0.17
87.3	2.70	12.53	0.94	0.19

Table S2 Double Debye parameters of Nafion 211

RH (%)	ε_∞	$\Delta\varepsilon_1$	$\Delta\varepsilon_2$	$\tau_2(ps)$
0	2.63	0.26	0.10	0.089
10	2.7	2.51	0.35	0.11
30	2.7	4.66	0.45	0.098
50	2.7	6.53	0.51	0.086
70	2.7	9.07	0.59	0.090
86.8	2.7	12.61	0.71	0.082

Table S3 Double Debye parameters of Nafion 211

RH (%)	ε_∞	$\Delta\varepsilon_1$	$\Delta\varepsilon_2$	$\tau_2(ps)$
0	2.65	0.16	0.10	0.078
10	2.7	1.43	0.25	0.13
30	2.7	3.84	0.39	0.11
50	2.7	5.39	0.45	0.10
70	2.7	7.59	0.52	0.098
90	2.7	10.74	0.65	0.11

Table S4 - Double Debye parameters of Nafion XL

Saturated salt measurement comparison

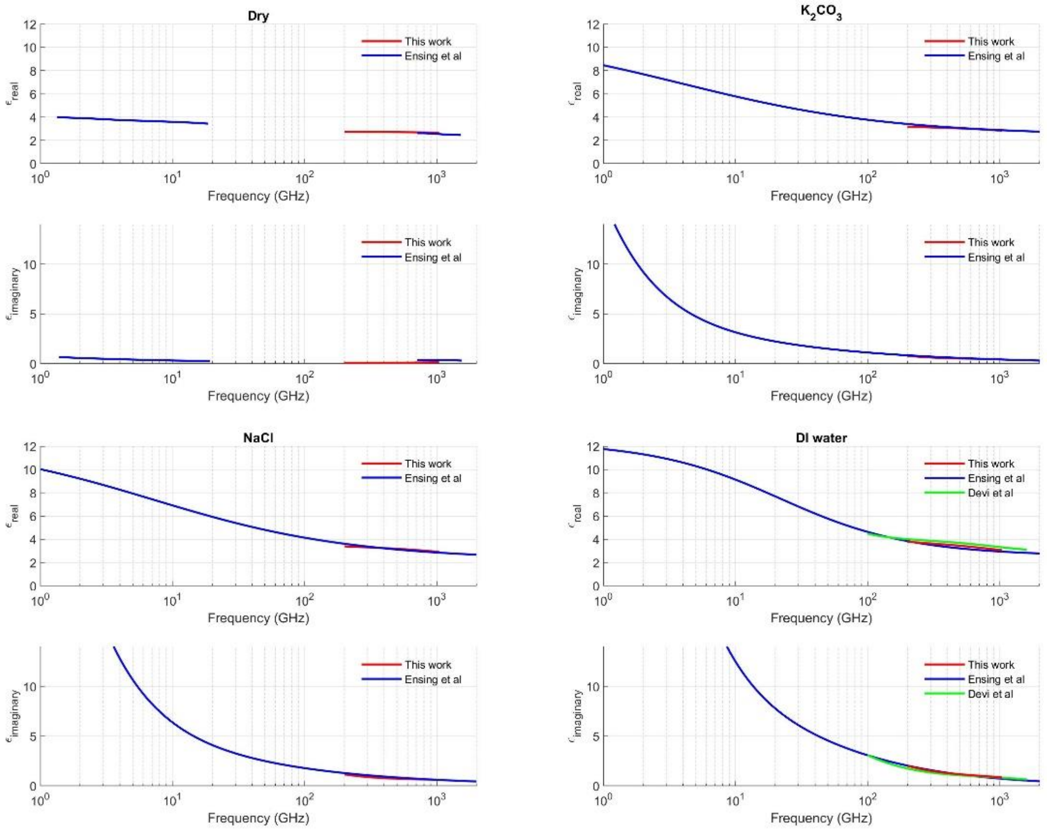

Figure S6 - Fitted permittivity comparison of Nafion 117 hydrated using saturated salts with literature.

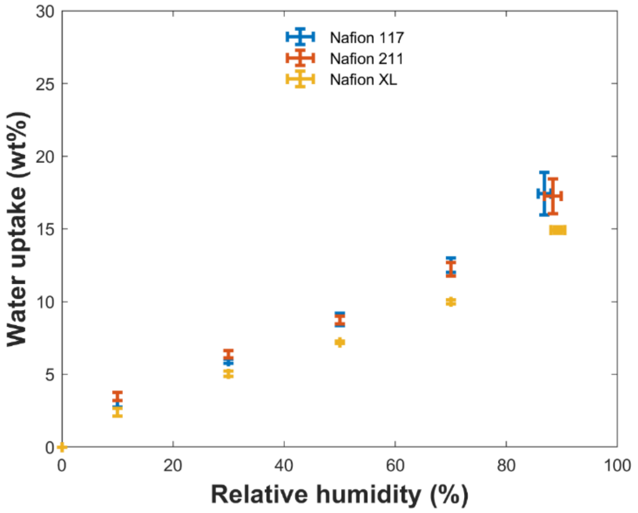

Figure S7 - Water uptake comparison of Nafion 117, Nafion 211 and Nafion XL

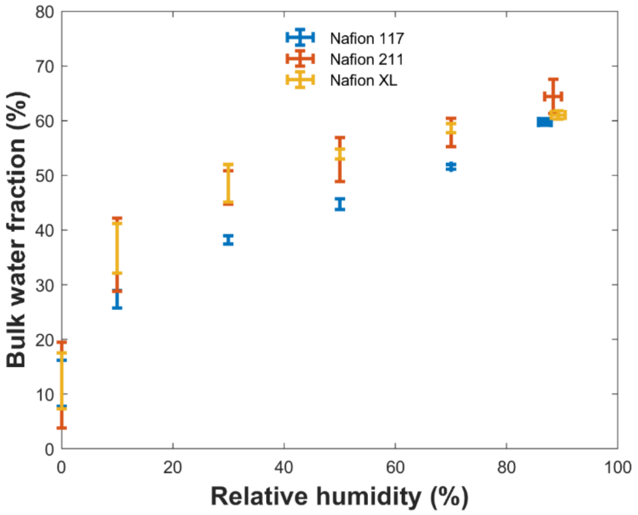

Figure S8 – Bulk water fraction comparison of Nafion 117, Nafion 211 and Nafion XL
